# Machine learning-based prediction of ICU admission and mortality in Crimean–Congo hemorrhagic fever by using wide-range targeted metabolomics

**DOI:** 10.1186/s12879-026-13787-5

**Published:** 2026-06-30

**Authors:** Ahu Cephe, Seyit Ali Büyüktuna, Necla Koçhan, Gözde Ertürk Zararsız, Serra İlayda Yerlitaş, Kübra Doğan, Demet Kablan, Gökhan Bağcı, Selda Özer, Cihad Baysal, Yasemin Çakır Kıymaz, Halef Okan Doğan, Gökmen Zararsız

**Affiliations:** 1 Institutional Data Management and Analytics Coordination Unit, Erciyes University, Kayseri, Türkiye; 2 Department of Infectious Disease and Clinical Microbiology, Cumhuriyet University School of Medicine, Sivas, Türkiye; 3 Department of Mathematics, Faculty of Arts and Sciences, Izmir University of Economics, İzmir, Türkiye; 4 Department of Biostatistics, School of Medicine, Erciyes University, Kayseri, Türkiye; 5 Department of Biochemistry, Minister of Health Sivas Numan Hospital, Sivas, Türkiye; 6 Department of Biochemistry, Cumhuriyet University School of Medicine, Sivas, Türkiye; 7 Department of Biochemistry, School of Medicine, Altinbas University, Istanbul, Türkiye; 8 Erciyes Teknopark, Hematainer Biotechnology and Health Products Inc., Kayseri, Türkiye

**Keywords:** Machine-learning, Crimean-Congo hemorrhagic fever, Survival modeling, Amino acids, Metabolomics

## Abstract

**Purpose:**

This study aimed to evaluate the potential of amino-acid profiles to predict disease progression in patients with Crimean–Congo Hemorrhagic Fever (CCHF) and to identify metabolic biomarkers associated with clinical outcomes and survival.

**Methods:**

Of the 115 confirmed CCHF patients, 18 required intensive care unit (ICU) admission and 16 died. Notably, 15 of the deaths occurred among ICU patients, whereas only one death occurred outside the ICU. For each patient, 32 amino acid concentrations were used as input for machine-learning (ML) models.

**Results:**

Among the classification models evaluated for predicting ICU admission, XGBOOST and LASSO achieved the highest performance, each with an AUC of 0.958. Arginine and glutamic acid consistently emerged as the most predictive features across all models, followed by 1-methyl-L-histidine, tryptophan, and tyrosine, which appeared among the top variables in four of the five best-performing models. In survival analysis, the mean concordance index (and integrated Brier score) was 0.973 (0.10) for Survival LASSO, 0.971 (0.11) for RFSRC, and 0.942 (0.12) for Survival XGBOOST. In survival models, the top five amino acids contributing to predictions were ornithine, gamma-aminobutyric acid, ethanolamine, arginine, and histidine.

**Conclusion:**

ML models based on amino-acid profiles can accurately predict disease progression in CCHF, supporting early risk stratification and providing insights into the metabolic mechanisms underlying disease severity.

**Supplementary Information:**

The online version contains supplementary material available at 10.1186/s12879-026-13787-5.

## Introduction

Crimean-Congo Hemorrhagic Fever (CCHF) is a severe and often fatal tick-borne viral disease caused by the Crimean–Congo hemorrhagic fever virus (CCHFV), classified within the Orthonairovirus haemorrhagiae species (genus Orthonairovirus, family Nairoviridae) [1]. It is endemic across a wide geographic region spanning Eastern Europe and the Middle East to Africa and Asia, with reported case-fatality rates from 10% to 40% [2]. Transmission occurs mainly through tick bites or contacts with infected animal or human body fluids [3]. The disease progresses through four phases—incubation, pre-hemorrhagic, hemorrhagic, and recovery [4, 5]—with early symptoms such as fever and malaise, and later complications including bleeding and multi-organ involvement [6, 7]. Due to its high pathogenicity, person-to-person transmissibility, and lack of specific treatment, CCHF remains a major public health threat and a potential bioterrorism concern [8].

In the absence of specific antiviral treatments or vaccines, early diagnosis using clinical and laboratory biomarkers associated with disease progression is crucial for improving survival in CCHF. Studies have demonstrated significant correlations between high viral load (>10^8^ copies/mL) and biomarkers such as deep thrombocytopenia, elevated AST and ALT levels, prolonged PT and aPTT, low fibrinogen, elevated ferritin levels, and clinical signs like hematemesis, melena, diarrhea, confusion, and somnolence [9–13]. Additional factors linked to poor prognosis include an increased neutrophil count, decreased lymphocyte and monocyte counts [12], elevated serum levels of pro-inflammatory cytokines [14], elevated NK (natural killer) cell count [15], heightened cytotoxic T cell levels [16], plasma cell-independent DNA [17], weak immune and antibody responses [18], a high DIC score [14], and increased cytotoxic T lymphocyte (CD8) counts [16]. Laboratory follow-up in surviving patients has shown improvements in leukopenia, hemostatic parameters, and renal function [18, 19]. Although numerous biomarkers have been identified, their combined ability to predict disease progression is limited, and the underlying pathogenic mechanisms remain unclear, as does the reason why CCHF manifests mildly in some cases but severely in others.

Growing evidence suggests that free amino acid profiling, which reflects key metabolic pathways, may provide valuable insights into disease diagnosis, monitoring, and prognosis. Altered amino acid profiles have been reported in viral and bacterial infections such as dengue [20] and COVID‑19 [21], as well as in clinical conditions including cancer [22], Alzheimer’s disease [23], and severe Crimean-Congo hemorrhagic fever [13, 24]. In CCHF, previous metabolomic studies, including our own exploratory work, identified significant alterations in amino acid pathways associated with disease severity and inflammation [25]. These findings suggest that metabolomic dysregulation may play an important role in CCHF pathogenesis.

Previous metabolomic analyses identified amino acid alterations associated with disease severity; however, their integration into predictive ML frameworks remains limited. Traditional statistical approaches are often insufficient to model the nonlinear, high-dimensional relationships inherent in metabolomic data for individualized risk forecasting. Machine learning (ML) methods offer an advanced analytical framework capable of capturing complex metabolic signatures, integrating multiple biomarkers simultaneously, and generating accurate, data-driven prognostic models [26–29]. Consequently, we hypothesized that traditional biochemical parameters may fall short in precisely forecasting the clinical trajectory of CCHF at an early stage, whereas ML algorithms trained on comprehensive amino acid profiles could overcome these limitations to support early clinical risk stratification and decision-making.

Given the substantial heterogeneity in clinical progression among CCHF patients, early identification of individuals at high risk for ICU admission or mortality remains a major clinical challenge. Building upon previously characterized metabolomic data, the present study focuses on predictive modeling and survival-based risk stratification using advanced machine learning algorithms applied to amino acid profiles. Therefore, this study aimed to develop, validate, and compare multiple machine learning approaches for predicting ICU admission and mortality in CCHF patients.

## Materials and methods

### Patients

This work is a pre-specified secondary analysis of a previously published prospective cohort study [25], in which baseline plasma free amino acid concentrations were quantified by LC–MS/MS in patients with CCHF. The earlier study [25] described the descriptive metabolic landscape of CCHF—comparing CCHF-positive, CCHF-negative symptomatic, and healthy control groups through univariate group comparisons, single-amino-acid ROC analyses, and pathway enrichment. The present study addresses a fundamentally different research question and does not duplicate any analysis reported in [25]. Specifically, we restrict the analytical population to the 115 RT-PCR/IgM-confirmed CCHF-positive patients and develop multivariable ML models for two prospectively recorded clinical endpoints—ICU admission and in-hospital all-cause mortality—neither of which was modeled in the prior publication.

No new patients were enrolled, and no new biological samples or amino acid measurements were generated for this analysis; the amino acid concentration matrix is identical to that reported in [25]. However, the clinical endpoint data used here—ICU admission status, time-to-event mortality data with exact admission and event/censoring dates, length of hospital stay, and ribavirin exposure stratified by clinical outcome group—were extracted de novo from medical records specifically for the present analysis and have not been previously published.

The definitive CCHF-positive status of all 115 included patients was molecularly and/or serologically confirmed at admission via positive real-time polymerase chain reaction (RT-PCR) and/or specific IgM enzyme-linked immunosorbent assay (ELISA) testing. Among the total cohort, 18 patients developed severe disease complications requiring intensive care unit (ICU) admission; all 18 of these ICU-admitted patients were confirmed CCHF-positive cases. Out of the 115 patients, a total of 16 patients died during follow-up, with 15 of these fatal cases occurring among the patients monitored in the ICU, whereas only one death occurred outside the ICU. Importantly, the prior publication [25] did not report ICU admission rates, mortality outcomes, or any time-to-event clinical data; these endpoints are reported here for the first time.

To document treatment-related variables that could potentially confound the ML modeling results, the distribution of off-label ribavirin use across outcome groups was specifically extracted for this study. No specific antiviral therapy for CCHF has been formally approved. From a clinical management perspective, although no standardized antiviral regimen exists, off-label oral or intravenous ribavirin therapy was administered to 20 patients (17.4% of the cohort), including 4 patients (22.2%) in the ICU group and 16 patients (16.5%) in the non-ICU group, with no statistically significant difference in distribution between clinical outcome groups (*p* = 0.589).

Unlike the prior study [25], which used the published Severity Grading Score (SGS) of Bakır et al. [30] as the severity outcome, the present analysis operationalized disease progression through two distinct prospectively recorded clinical endpoints to evaluate the predictive capacity of the amino acid models. First, for binary classification algorithms, ‘severe disease progression’ was explicitly defined as the clinical necessity for intensive care unit admission during hospital follow-up. Second, for time-to-event survival models, the endpoint was strictly defined as all-cause in-hospital mortality, tracked from the exact date of baseline hospital admission to death or discharge.

The study was conducted in accordance with the Declaration of Helsinki and approved by the Ethics Committee of Cumhuriyet University (approval number: 2023–11/22). Written informed consent was obtained from all participants or their legal guardians.

### Sample collection and amino acid analysis

Blood samples were collected from all participants at the time of hospital admission (baseline), prior to any clinical deterioration or intensive care unit transfer. Amino acid concentrations were quantified using liquid chromatography-tandem mass spectrometry (LC-MS/MS) using the validated commercial Jasem Quantitative Amino Acids Kit on an Agilent 6470 triple-quadrupole platform, exactly as previously described in [25]. In this study, a targeted metabolomics approach was employed, and a total of 32 amino acids were measured for each patient, including both proteinogenic and non-proteinogenic amino acids. The 32-amino-acid panel covers biologically relevant pathways implicated in CCHF pathogenesis (urea cycle, macrophage polarization, inflammation, and hepatic metabolism) and corresponds to the analytically validated panel of the commercial kit used in [25]. The amino acid concentration matrix used as input for all ML analyses in the present study is the same matrix reported in [25]; no re-measurement was performed.

### Statistical analysis and machine learning models

Data normality was assessed using histograms, Q–Q plots, and the Shapiro–Wilk’s test, while homogeneity of variances was evaluated using Levene’s test. Prior to model training, the missing data profile was rigorously evaluated. The overall missingness across the 32 quantified amino acids was exceptionally low (<2%) and followed a Missing Completely at Random (MCAR) pattern. A comprehensive breakdown of the exact missing value numbers and percentages for each individual metabolite is detailed in Supplementary Table S2. To prevent any form of data leakage, missing values were imputed using an iterative, non-parametric Random Forest (RF) imputation algorithm, executed strictly and dynamically within each cross-validation training fold. To compare differences between study groups in clinical, laboratory, and amino acid concentration variables, a two-sided independent samples t-test or the Mann–Whitney U test was applied for continuous variables, depending on the distribution. For categorical variables, Fisher’s exact test or the Pearson chi-square test was used. Categorical variables were presented as frequencies and percentages, while continuous variables were expressed as means ± standard deviations for normally distributed data, and as medians with interquartile ranges (1st–3rd quartiles) for non-normally distributed data.

ML models were used to predict disease progression in CCHF patients. For the prediction model building process, a series of preprocessing steps were first applied to the amino acid concentration data. To evaluate the generalizability and robustness of the models, the dataset was randomly split into training and test sets, using a 70:30 ratio. To reduce potential bias due to variability in train–test partitioning, analyses were conducted across 30 independent training–test splits. The training sets were used for model training and hyperparameter tuning, while the test sets were reserved for performance evaluation (Supplementary Table S3). To minimize overfitting in the ML models and to identify the most informative predictors among the 32 amino acids, two different feature selection methods were applied to the training set: recursive feature elimination (RFE) [31] and Boruta feature selection (Boruta) [32]. To prevent bias toward the majority class during model training, the data distribution was reconstructed and balanced using the synthetic minority oversampling technique (SMOTE) [33], as well as upsampling (UP) and downsampling (DOWN) technique.

After completing the preprocessing steps mentioned above, the model development phase was initiated. To address the classification problem of predicting disease progression in CCHF patients, six ML models were applied: Random Forest, Least Absolute Shrinkage and Selection Operator (LASSO), K-Nearest Neighbors (KNN), Nearest Shrunken Centroid (NSC), Support Vector Machine (SVM), and eXtreme Gradient Boosting (XGBOOST). These models were implemented under two main conditions: datasets that were either balanced or imbalanced, and the application of feature selection methods. Prior to classification model development, feature selection was performed using both RFE and the Boruta methods, resulting in a total of 48 distinct classification scenarios. To address the survival problem of predicting mortality in CCHF patients, Random Survival Forest (RFSRC), Survival LASSO, and Survival XGBOOST were employed. Following model training, variable importance analysis was performed to rank each amino acid’s contribution to predicting disease progression, identifying those most strongly associated with intensive care need.

To prevent data leakage and ensure methodological rigor, all preprocessing procedures, including missing value imputation, feature selection (Boruta and RFE), and class balancing techniques (SMOTE, upsampling, and downsampling), were performed exclusively within the training folds during the cross-validation process. Hyperparameter optimization was conducted using repeated 5-fold cross-validation with grid search applied only to the training data. The independent test set was completely isolated from all preprocessing and model training procedures and was used solely for final model evaluation. This nested analytical framework was implemented to avoid optimistic bias and preserve the generalizability of model performance estimates.

For time-to-event outcomes, survival analysis was modeled from the exact date of hospital admission (t0) to the date of death (event) or hospital discharge (right-censoring). The follow-up timeline focused strictly on the acute in-hospital duration, capturing the critical phase of CCHF progression. Patients who survived and were clinically discharged without further complications were right-censored at their total length of hospital stay. Given the limited number of fatal events (*n* = 16), complex survival architectures such as RFSRC and Survival LASSO were implemented with stringent internal precautions to safeguard against overfitting. Specifically, hyperparameter tuning and model training for all survival algorithms were performed strictly using a nested 5-fold cross-validation scheme with 10 independent repetitions, ensuring that out-of-bag prediction paths and penalty parameters (λ selection in LASSO) were validated on isolated folds before computing the final Concordance Indices and Integrated Brier Scores (IBS).

To enhance robustness and minimize overfitting, a five-fold cross-validation with 10 repetitions was applied exclusively to the training datasets. The predictive performance of the classification models was evaluated on the test set using area under curve (AUC), accuracy (ACC), sensitivity (SENS), specificity (SPEC), positive predictive value (PPV), negative predictive value (NPV), positive likelihood ratio (LR+), and negative likelihood ratio (LR−). Also, to visualize the predictiveness performance of prediction models, predictiveness curves were drawn using R’s predictiveness_curve package to visualize overall discriminative performance [34]. For survival models, Harrell’s concordance index (C-index), IBS, and D-calibrated metrics were used for performance assessment on the test set. Higher AUC and ACC values indicate better classification performance in predicting patient disease progression status. Similarly, higher values of SENS, SPEC, PPV, NPV, and LR+, along with lower LR− values, reflect stronger classification performance. In survival analysis, superior model performance is demonstrated by higher C-index and D-calibrated values, alongside lower IBS values. After performance metrics were obtained separately on each of the 30 different training and test sets, the average value was calculated for each metric.

Adjusted *p*-values were calculated using the Benjamini–Hochberg procedure [35] to correct for multiple testing. Adjusted *p*-values less than 0.05 were considered statistically significant. All statistical analyses were conducted using R software version 4.3.1 (http://www.R-project.org/).

## Results

### Patient characteristics

The demographic and clinical characteristics of the patient cohort, stratified by the ICU admission endpoint specific to the present analysis, are summarized in Table 1. While the same 115 CCHF-positive patients were described in [25] under a different stratification (positivity status across three groups), Table 1 of the present manuscript presents these baseline variables for the first time under the ICU-versus-non-ICU stratification used for ML model development. In line with the demographic homogeneity of the overall cohort reported in [25], no statistically significant differences in age or gender were observed between CCHF-positive patients who required ICU admission and those who did not in the present ICU-stratified analysis. However, consistent with previous findings and the established literature on severe CCHF pathogenesis [25], patients requiring ICU admission exhibited marked hematologic and hepatic dysfunction. This was characterized by significantly elevated levels of white blood cells (WBC#), neutrophils (NEUT#), immature granulocytes (IG#, IG%), creatinine, amylase, ALP, ALT, AST, LDH, CK, and GGT, alongside significantly lower RBC, HGB, PLT, total protein, and albumin levels. Additionally, 83.3% of the deceased patients required ICU admission. These characterized clinical and laboratory baseline features confirm the clinical severity of the cohort, providing a robust clinical dataset to train and validate our prognostic machine learning models.

The definitive CCHF-positive status of all 115 included patients was molecularly and/or serologically confirmed at admission via positive real-time polymerase chain reaction (RT-PCR) and/or specific IgM enzyme-linked immunosorbent assay (ELISA) testing. Among the total cohort, 18 patients developed severe disease complications requiring intensive care unit (ICU) admission; all 18 of these ICU-admitted patients were confirmed CCHF-positive cases. Out of the 115 patients, a total of 16 patients died during follow-up, with 15 of these fatal cases occurring among the patients monitored in the ICU, whereas only one death occurred outside the ICU. Importantly, the prior publication [25] did not report ICU admission rates, mortality outcomes, or any time-to-event clinical data; these endpoints are reported here for the first time.

**Table 1:** Demographic and clinical characteristics of the patients

Variables	ICU admission		Total (n = 115)	Adj. p-value
	Yes (n = 18)	No (n = 97)		
Age	57.61 ± 17.76	54.96 ± 16.53	55.37 ± 16.67	0.587
Gender (Female)	9 (50.0)	37 (38.1)	46 (40.0)	0.402
WBC	6.73(4.54–14.66)	3.06(2.17–4.55)	3.22(2.23–4.96)	**0.002**
NEUT#	5.62(2.90–12.63)	2.14(1.26–3.39)	2.39(1.30–3.99)	**0.002**
LYMPH#	0.74(0.36–1.95)	0.57(0.37–0.74)	0.57(0.37–0.85)	0.111
MONO#	0.12(0.06–0.40)	0.20(0.14–0.35)	0.19(0.12–0.35)	0.136
RBC	4.47 ± 0.79	4.85 ± 0.57	4.79 ± 0.62	**0.023**
HGB	12.91 ± 2.37	14.23 ± 1.79	14.02 ± 1.94	**0.012**
HCT	38.42 ± 6.85	41.52 ± 4.81	41.03 ± 5.27	0.111
PLT	39.94 ± 30.90	104.58 ± 51.45	94.46 ± 54.11	**0.002**
IG#	0.67(0.16–1.94)	0.02(0.01–0.04)	0.03(0.01–0.07)	**0.002**
IG%	8.95(5.08–14.20)	0.70(0.45–1.20)	0.80(0.50–1.80)	**0.002**
Creatinine	1.64(0.90–3.68)	0.92(0.79–1.11)	0.94(0.80–1.19)	**0.002**
T. PROT	61.28 ± 5.21	65.83 ± 5.87	65.12 ± 5.99	**0.006**
Albumin	35.82 ± 4.35	39.92 ± 3.87	39.28 ± 4.21	**0.002**
Amylase	117.50(86.25–189.75)	77.00(61.00–101.00)	81.00(63.00–110.00)	**0.002**
ALP	136.00(90.25–271.25)	72.00(62.50–95.00)	77.00(64.00–99.00)	**0.002**
ALT	119.00(43.50–497.75)	47.00(23.00–83.00)	50.00(25.00–90.00)	**0.007**
AST	455.00(161.50–1342.00)	72.00(37.00–137.50)	82.00(44.00–187.00)	**0.002**
LDH	1193.00(567.25–2415.50)	366.00(265.50–526.00)	383.00(272.00–582.00)	**0.002**
CK	558.50(255.50–1358.75)	231.00(125.50–581.00)	284.00(138.00–626.00)	**0.020**
GGT	146.50(34.00–274.25)	30.00(17.50–67.00)	35.00(19.00–89.00)	**0.002**
Lipase	72.00(47.75–95.75)	49.00(35.50–86.50)	54.00(37.00–88.00)	0.090
**SGS Groups**				
Low	1 (5.6)^a^	81 (83.5)^b^	82 (71.3)	**<0.001**
Medium	6 (33.3)^a^	16 (16.5)^a^	22 (19.1)	
High	11 (61.1)^a^	0 (0.0)^b^	11 (9.6)	
Exitus (Yes)	15 (83.3)	1 (1.0)	16 (13.9)	**0.002**
Phase (Haemorrhagic)	11 (61.1)	9 (9.3)	20 (17.4)	**0.002**
**Comorbidities**				
Diabetes mellitus (Yes)	0 (0.0)	6 (6.2)	6 (5.2)	0.334
Hypertension (Yes)	4 (22.2)	9 (9.3)	13 (11.3)	0.143
Chronic obstructive pulmonary disease (Yes)	3 (16.7)	8 (8.2)	11 (9.6)	0.329
Coronary artery disease (Yes)	1 (5.6)	6 (6.2)	7 (6.1)	0.918
Malignancy (Yes)	0 (0.0)	1 (1.0)	1 (0.9)	0.867
Chronic kidney failure (Yes)	0 (0.0)	0 (0.0)	0 (0.0)	
Rheumatic disease (Yes)	1 (5.6)	3 (3.1)	4 (3.5)	0.561
**Drugs**				
Ribavirin (Yes)	4 (22.2)	16 (16.5)	95 (82.6)	0.589
Steroid (Yes)	18 (100.0)	34 (35.1)	52 (45.2)	**0.002**
TDP (Yes)	18 (100.0)	59 (60.8)	77 (67.0)	**0.002**
Thrombocytes (Yes)	17 (94.4)	40 (41.2)	57 (49.6)	**0.002**
Use of antibiotic (Yes)	11 (61.1)	26 (26.8)	37 (32.2)	**0.007**

Adj.p: Adjusted p-values using Benjamini-Hochberg procedure, ALP: Alkaline phosphatase, ALT: Alanine amino transferase, AST: Aspartate amino transferase, CK: Creatinine kinase, GGT: Gamma glutamyl transferase, HCT: Hematocrit, HGB: Hemoglobin, IG#: Immature granulocyte count, IG%: Immature granulocyte percentage, LDH: Lactate dehydrogenase, LYMPH#: Lymphocyte count, MONO#: Monocyte count, NEUT#: Neutrophile count, PLT: Platelet, RBC: Red blood cell, T. PROT: Total protein, WBC: White blood cell count, SGS: Severity Grading Score. The values are demonstrated as mean±standard deviation, median (1st-3rd quartiles) or n (%). Statistically significant adjusted p-values are shown in bold

### Machine learning model performance for ICU admission prediction

Before training the ML models, the RFE and Boruta methods were applied to identify the most important amino acid predictors for disease progression in CCHF-positive patients. Supplementary Figure S1-2 display the frequency table showing how many times each variable was selected by the RFE and Boruta methods across 30 trials. Using the RFE method, gamma-aminobutyric acid was selected in every trial. Additionally, ornithine, tyrosine, arginine, ethanolamine, and glutamic acid were selected by RFE method in 20 to 30 trials. According to the Boruta method, tyrosine, ornithine, 1-methyl-l-histidine, methionine, histidine, glutamic acid, gamma-aminobutyric acid, ethanolamine, and arginine were consistently selected in all trials. Phenylalanine, lysine, glutamine, alanine, cysteine, 3-aminoisobutyric acid, and tryptophan were selected in 20 to 30 trials. Amino acids identified by both the RFE and Boruta methods in over 20 trials, as shown in Supplementary Figure S3, included arginine, ethanolamine, tyrosine, gamma-aminobutyric acid, ornithine, and glutamic acid.

**Fig. 1:**
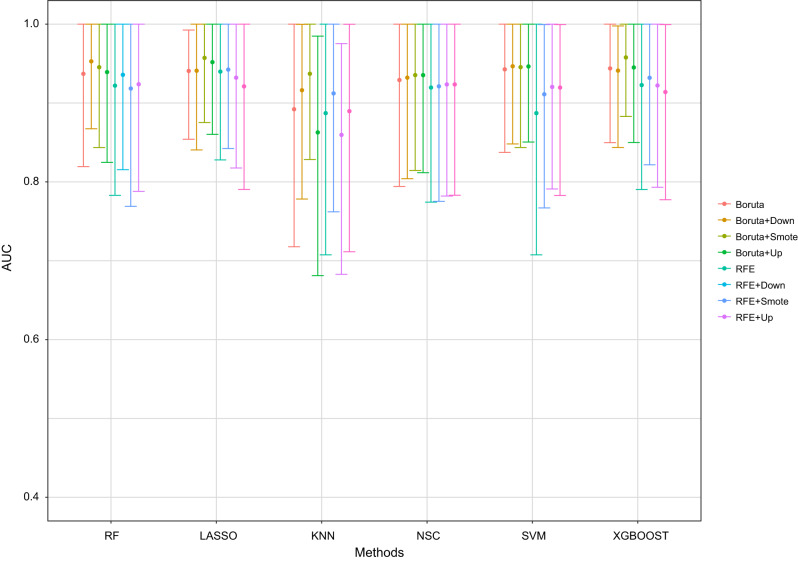
Predictive performance comparison (area under the curve - AUC) of six machine learning models for predicting ICU admission in CCHF patients. The performance metrics represent the mean AUC values evaluated over 30 independent training-test splits (70:30 ratio). The x-axis lists the six evaluated classification architectures: RF, LASSO, KNN, NSC, SVM, and XGBOOST. The y-axis shows the AUC score ranging from 0.4 to 1.0. Error bars denote the confidence intervals across trials. Color coding represents the combinations of feature selection methods (RFE; Boruta) and class balancing/sampling algorithms (SMOTE; UP; DOWN)

**Fig. 2:**
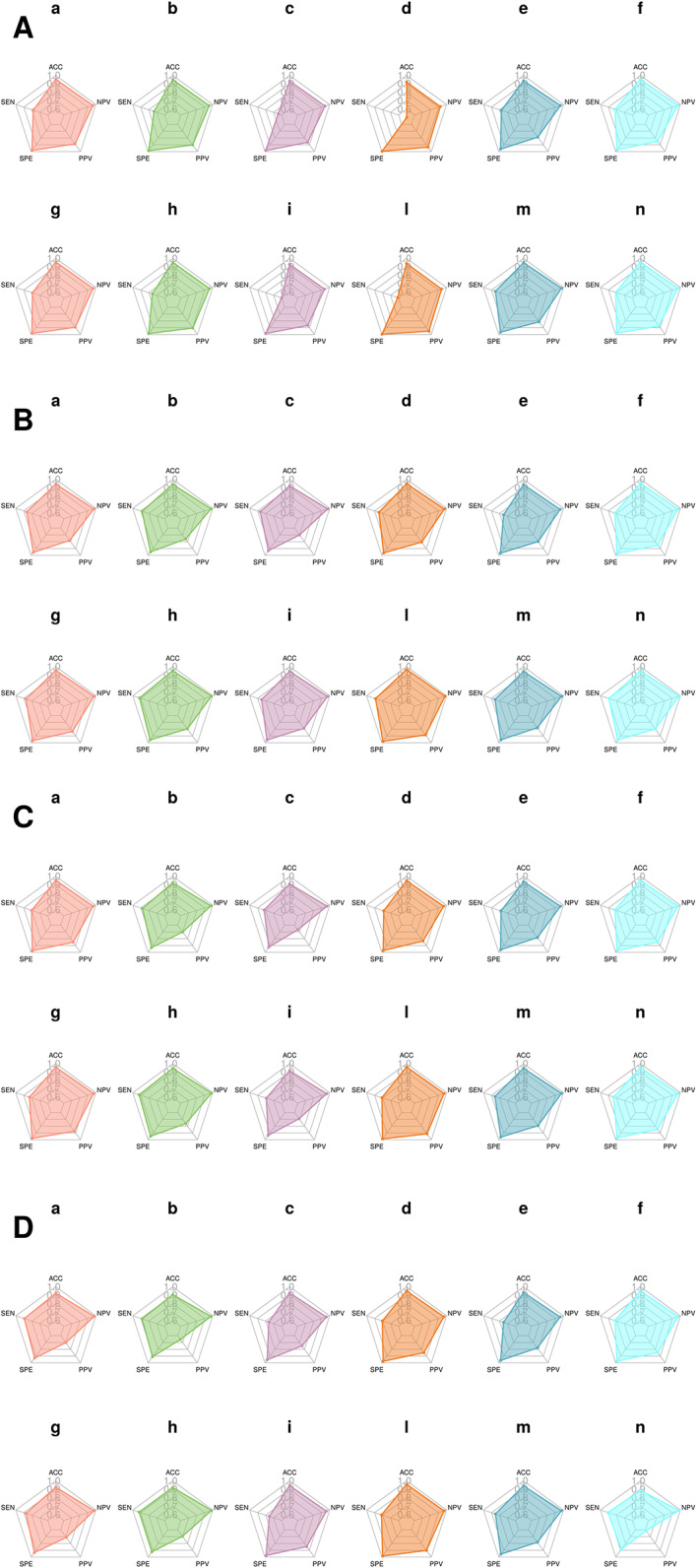
Radar charts demonstrating multivariate classification performance metrics across 48 distinct machine learning scenarios. Panels A, B, C, and D represent the baseline imbalanced dataset, SMOTE-balanced, UP-sampled, and DOWN-sampled datasets, respectively. Individual radar charts (a to n) display five key validation metrics: ACC, SENS, SPEC, PPV, and NPV. Models are split by feature selection methods: (a–f) utilize RFE, and (g–n) utilize Boruta feature selection across the six classifiers (RF, LASSO, KNN, NSC, SVM, XGBOOST)

Figure 1 and Supplementary Table S1 depict the mean AUC values of ML models used to classify whether CCHF-positive patients who required ICU admission. The results are shown separately for each classification model, evaluated using two feature selection methods (RFE and Boruta) and three data balancing techniques (SMOTE, UP, and DOWN). Combining Boruta method with the SMOTE technique resulted in the highest average AUC values, especially for the XGBOOST and LASSO models (both AUC = 0.958). The RF model combined with Boruta method with the DOWN technique also demonstrated high performance (AUC = 0.953). Similarly high performance was achieved by LASSO, SVM, and XGBOOST when applying Boruta method with the UP technique (AUCs = 0.952, 0.947, and 0.946, respectively). The KNN model with RFE method and the UP technique yielded the lowest AUC (0.86).

ACC, SENS, SPEC, PPV, and NPV values for all classification models are provided in Supplementary Table S1 and Fig. 2 (A-D). The highest ACC value was observed in the NSC model when Boruta method was combined with the SMOTE technique (ACC = 0.958). The application of sampling algorithms improved ACC values in both the NSC and KNN models. For example, the ACC of the NSC model with Boruta method on the imbalanced dataset increased from 0.924 to 0.958 with SMOTE, to 0.951 with UP, and to 0.950 with DOWN. Sampling techniques also led to improvements in SENS and NPV values. For instance, in the NSC model with RFE method, the SENS value increased from 0.374 on the imbalanced data to 0.807 with SMOTE.

**Fig. 3:**
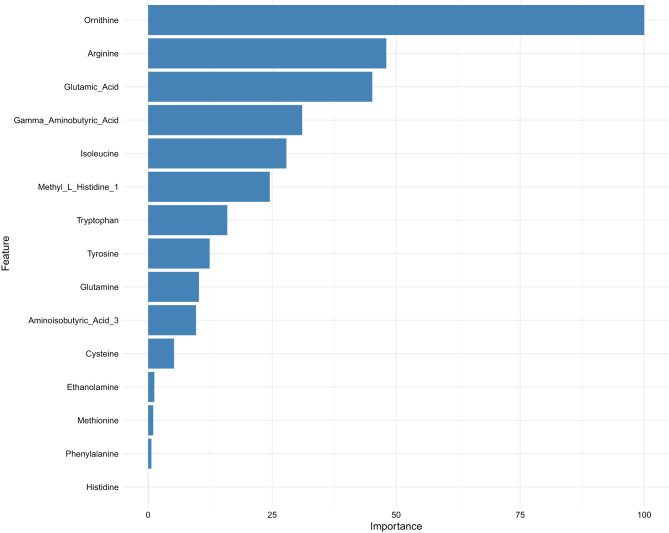
Feature importance plot for the top-performing XGBOOST model combined with Boruta feature selection. The y-axis ranks the most influential amino acid biomarkers predicting disease progression (ICU admission). The x-axis quantifies the relative variable importance score normalized to a scale of 0 to 100

**Fig. 4:**
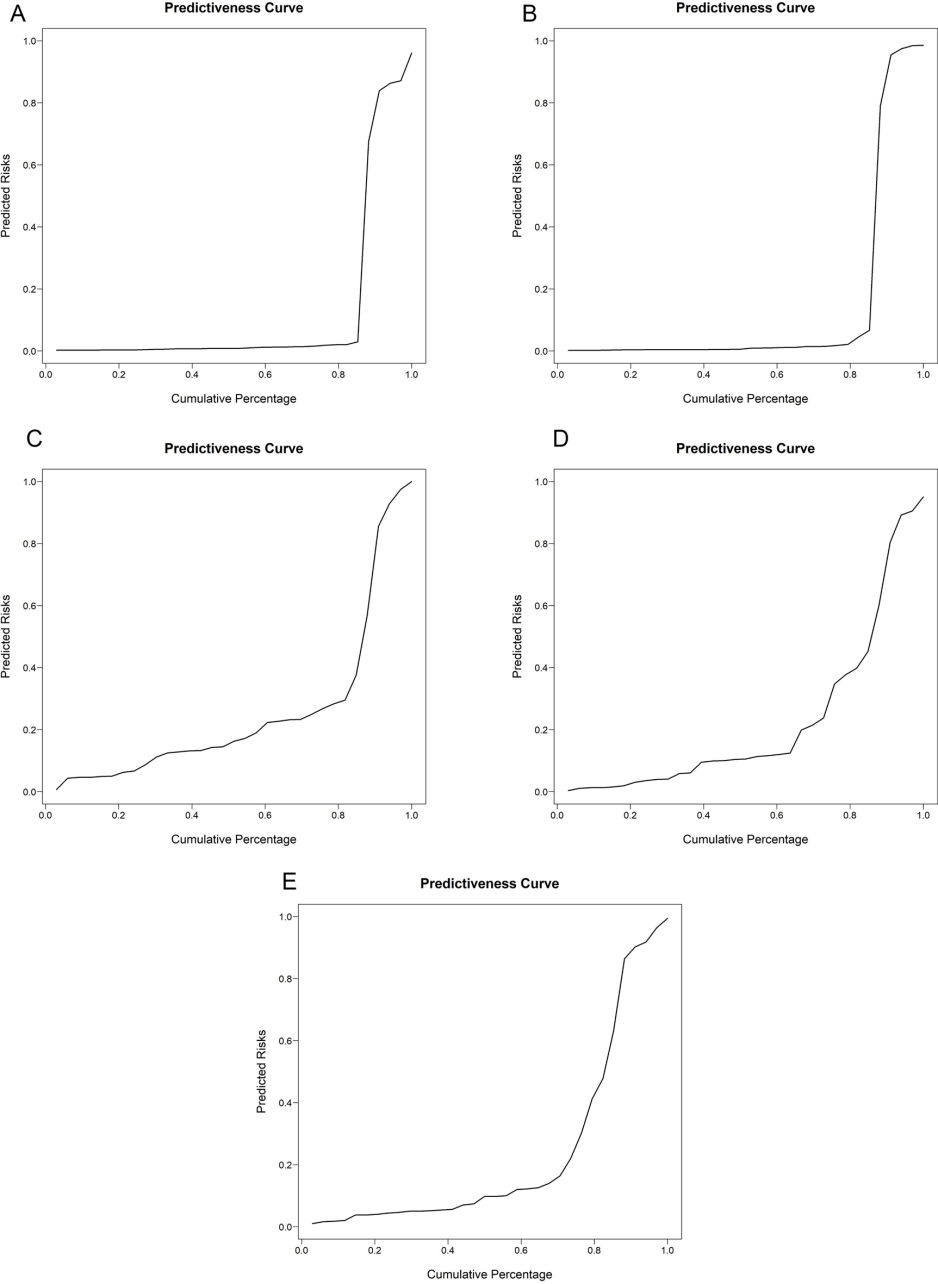
Predictiveness curves evaluating overall discriminative power for the top five high-AUC machine learning models. Panels display: (**A**) XGBOOST+Boruta, (**B**) XGBOOST+Boruta+SMOTE, (**C**) LASSO+Boruta+SMOTE, (**D**) LASSO+Boruta+UP, and (**E**) RF+Boruta+DOWN. The x-axis represents the cumulative percentage of the patient population, and the y-axis shows the predicted probability risks for severe disease progression (ICU admission)

When the top five models with the highest AUC values mentioned above were evaluated together, arginine and glutamic acid emerged as the most critical metabolic drivers for predicting disease progression across all optimized models, followed by 1-methyl-L-histidine, tryptophan, and tyrosine (Fig. 3, Supplementary Figure S4-6). It is highly noteworthy that while previous univariate analysis [25] highlighted a widespread amino acid, the multi-variable machine learning framework successfully pruned this redundant dimensionality, proving that a select signature of just 3 to 5 key biomarkers holds the actual predictive power. For instance, tryptophan, which did not stand out significantly in traditional univariate testing, emerged here as a top prognostic feature due to its complex non-linear interactions captured by the XGBOOST and RF algorithms. This underscores the superiority of ML in identifying latent metabolic predictors that traditional statistical profiling overlooks. These results are broadly consistent with the directionality of amino-acid alterations previously described in [25], with the notable exception of tryptophan—which did not stand out in single-marker analyses but emerged as a top predictor under the multivariable ML framework due to its non-linear interactions captured by XGBOOST and RF. Predictiveness curves for these high-AUC models are shown in Fig. 4. According to these curves, the XGBOOST model developed with Boruta demonstrated the highest discriminative power in predicting disease progression.

**Fig. 5:**
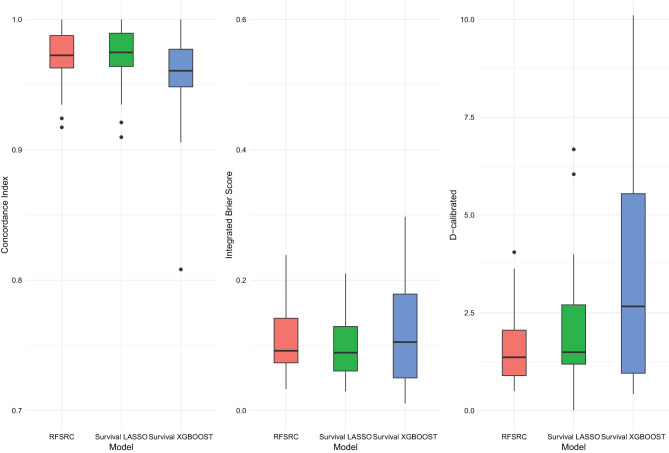
Performance assessment of the three survival machine learning models for predicting CCHF mortality. Box plots demonstrate the distribution of results across cross-validation runs for three performance metrics: concordance index (higher is superior), integrated Brier score (lower is superior), and D-calibrated metric. The evaluated algorithms on the x-axis are RFSRC, survival LASSO, and survival XGBOOST

**Fig. 6:**
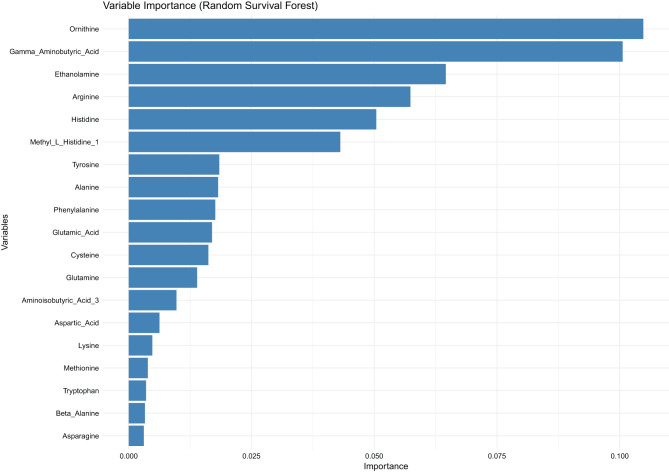
Variable importance ranking derived from the RFSRC model. The plot displays the top-ranked plasma amino acids contributing to the prediction of overall survival/mortality in CCHF patients. The x-axis reflects the relative importance score

### Survival modeling with amino acids concentration for CCHF

Prior to survival modeling, the Boruta method was applied and identified the following key variables: ornithine, gamma-aminobutyric acid, ethanolamine, arginine, histidine, 1-methyl-L-histidine, tyrosine, alanine, phenylalanine, glutamic acid, cysteine, glutamine, 3-aminoisobutyric acid, aspartic acid, lysine, methionine, tryptophan, beta-alanine, and asparagine.

The mean concordance index was 0.973 for Survival LASSO, 0.971 for RFSRC, and 0.942 for Survival XGBOOST. The integrated Brier scores were 0.10, 0.11, and 0.12 for Survival LASSO, RFSRC, and Survival XGBOOST, respectively. Calibration values were 2.13 for Survival LASSO, 1.61 for RFSRC, and 7.78 for Survival XGBOOST (Fig. 5). In the scenario where the RFSRC model yielded the best performance, ornithine and gamma-aminobutyric acid emerged as the two most important variables (Fig. 6).

## Discussion

This study demonstrates that machine learning models based on amino acid profiles can accurately predict disease progression in CCHF patients, achieving AUC values exceeding 0.95 for ICU admission prediction and C-index of 0.973 for survival prediction. To our knowledge, this is the first study to systematically apply machine learning to amino acid profiling for prognostic stratification in CCHF.

Among the evaluated classifiers, XGBOOST and LASSO consistently outperformed other models, achieving AUC values close to 0.96 for ICU prediction. These results are consistent with previous studies showing that ensemble and regularized methods are well suited for high-dimensional biomedical data with limited sample sizes [36, 37]. Although RF and SVM also achieved high performance (AUC = 0.95), their sensitivity to hyperparameter selection and class imbalance may limit robustness in heterogeneous clinical settings [38, 39]. In contrast, distance-based methods such as KNN exhibited lower performance, likely due to overlapping class distributions and the curse of dimensionality [39]. Overall, the results indicate that model choice plays a critical role in extracting clinically meaningful patterns from metabolic data.

Given the pronounced class imbalance characteristic of clinical datasets, particularly in severe infectious diseases where adverse outcomes are relatively rare, we implemented multiple sampling strategies (SMOTE, UP, and DOWN) to mitigate bias and enhance model reliability. These approaches substantially improved sensitivity, a clinically critical metric for early identification of high-risk patients. Notably, XGBOOST with Boruta feature selection exhibited a baseline sensitivity of 0.76, which increased to 0.794 with UP, 0.867 with SMOTE, and 0.88 with DOWN. Similarly, LASSO sensitivity improved from 0.694 at baseline to 0.894 with SMOTE and 0.90 with UP and DOWN. For KNN, SMOTE increased overall performance by 4.5%, with marked gains in accuracy (1.7%), sensitivity (31.4%), and negative predictive value (4.8%). These findings highlight the necessity of explicit imbalance handling in clinical ML applications to achieve clinically meaningful performance. This technical optimization demonstrates that combining robust multi-variable feature pruning with synthetic minority oversampling is essential for building reliable prognostic tools from high-dimensional metabolomic data, a methodological advancement that extends far beyond traditional univariate statistical analyses.

Numerous studies with different study designs conducted in various regions of the world have defined case criteria for CCHF and reported prognostic factors based on the evaluation of epidemiological data, clinical findings, and laboratory test results [13, 40, 41]. Diagnostic confirmation relies on PCR detection of viral RNA or serological identification of CCHFV-specific IgM antibodies [42]. Although clinical scoring systems such as the Severity Grading Score (SGS) [30, 43] and Severity Scoring Index (SSI) [24] facilitate early risk assessment, these models are predominantly driven by clinical observations and routine laboratory measurements. In contrast, our approach uses amino acid metabolic profiles that directly reflect underlying molecular perturbations associated with disease severity, enabling more sensitive discrimination of patients requiring intensive care.

The use of amino acid profiling to predict ICU requirements in patients with CCHF offers several advantages over traditional epidemiological data, clinical findings, and routine laboratory tests. Notably, metabolic alterations frequently precede overt clinical deterioration, thereby providing an early window for ICU triage [44, 45, 46]. In this context, disruptions in arginine and citrulline metabolism reflect urea cycle dysfunction and have been associated with increased disease severity [47]. Beyond their temporal advantage, amino acids are directly involved in key pathogenic processes, including inflammation, oxidative stress, immune regulation, hepatic dysfunction, and coagulopathy [45, 47]. From a methodological perspective, amino acid measurements obtained using LC–MS or targeted metabolomics are quantitative, objective, and highly reproducible, in contrast to the inherently subjective nature of certain clinical evaluations. Moreover, metabolic profiling captures disease-specific biochemical signatures, such as the arginine–citrulline imbalance observed in CCHF [48], which is also critical in CCHF progression [25]. Ultimately, amino acids serve not only as prognostic biomarkers but also as potential therapeutic targets, thereby offering an integrated framework for both risk stratification and intervention.

Büyüktuna et al. [25] previously characterized the descriptive amino-acid landscape of CCHF using univariate approaches [25]. The present multivariable ML framework moves beyond that descriptive stage by integrating the full 32-amino-acid panel and capturing non-linear interactions, which likely accounts for the superior predictive accuracy achieved here compared with single-metabolite assessments.

Delayed diagnosis may result in life-threatening complications, including hemorrhage, coagulopathy, shock, and multi-organ failure. Therefore, early diagnosis of CCHF is of critical importance for reducing mortality and morbidity, enabling early prediction of ICU requirements, ensuring timely initiation of appropriate supportive therapy, preventing nosocomial and community transmission, and optimizing the efficient use of healthcare resources. In this context, even a small improvement in model performance can have significant clinical implications, as it may allow for earlier identification of high-risk patients, more accurate allocation of ICU resources, and timely intervention to prevent severe complications, ultimately contributing to better patient outcomes and more efficient healthcare delivery.

The modeling process enabled the identification of key amino acid biomarkers with prognostic value for disease progression. In the best-performing classification model, the top five variables contributing most substantially to model performance were ornithine, arginine, glutamic acid, GABA, and isoleucine, whereas for survival modeling, ornithine, GABA, ethanolamine, arginine, and histidine emerged as the most influential features. The prominence of arginine, histidine, and glutamic acid is consistent with findings reported in previous study [25], supporting their established role in CCHF-related metabolic dysregulation. While the individual significance of these amino acids was previously identified, the present study establishes for the first time their combined prognostic utility through feature selection and variable importance analyses in validated predictive models. Additionally, the identification of novel biomarkers such as ethanolamine highlights the added value of the present approach and suggests previously underexplored metabolic pathways that may be involved in disease severity and outcomes. From this perspective, the findings of this study not only reinforce existing evidence but also provide a foundation for future research aimed at elucidating novel prognostic biomarkers and therapeutic targets in CCHF.

A marked differential abundance of arginine (lower) and ornithine (higher) was observed between ICU-requiring and non-ICU CCHF patients, and arginine emerged as a statistically significant variable across all predictive models. While [25] previously described the overall arginine–ornithine imbalance in CCHF descriptively, the present ML framework moves from descriptive discovery to predictive application by quantifying the prognostic utility of this axis at the individual-patient level. Arginine and ornithine are intermediate metabolites of the urea cycle and shape the inflammatory response through M1–M2 macrophage polarization [49]. The enzyme arginase, released by M2 macrophages, converts L-arginine to ornithine, reducing nitric oxide production; this mechanism mediates processes such as immunosuppression, tissue repair, fibrosis, and suppression of T-cell responses. This process has been examined mainly in the context of cancer and other chronic inflammatory conditions in the literature, and studies on its metabolic responses in infectious diseases are still limited [50–56]. Accordingly, we think that the shift from M1 to M2 phenotype in CCHF may have facilitated viral replication by increasing immune suppression. Previous studies have shown that the levels of cytokines associated with M2 macrophages, including IL-10, IL-4 and TGF-β1, increase in CCHF patients and this increase is associated with severe clinical course [14, 57–59]. These data support our hypothesis that the change in arginine-ornithine balance observed in our study may be related to M2 macrophage polarization. In this context, strategies to shift macrophage polarization back to the M1 direction or to inhibit arginase-1 can be considered as potential new targets in the treatment of CCHF.

It was previously shown that ethanolamine is elevated in CCHF patients compared with healthy controls and correlates positively with hepatic enzymes. In the present study, ethanolamine ranked among the top-tier survival predictors in the RFSRC model. Ethanolamine is one of the key precursors for phosphatidylethanolamine synthesis in the liver, playing a critical role in the production of PE, the building block of cell membranes [61]. CCHF is an acute viral infection characterized by high fever, widespread bleeding tendency, and severe liver dysfunction, causing extensive liver damage [60]. It was concluded that it was caused by the release of ethanolamine, a precursor of phosphatidylethanolamine, which could not be synthesized, into plasma as a result of a disruption in the phosphatidylethanolamine synthesis pathway due to liver damage, and that this increase was directly related to both the severity of liver damage and the clinical severity of the disease.

While this study provides robust evidence for the prognostic value of amino acid profiling, certain limitations warrant consideration. First, the single-center design may restrict the generalizability of our predictive models across different geographic regions or viral genotypes. Additionally, although the models were rigorously validated through internal cross-validation and independent test splits, they currently lack external validation. Prospective studies on larger, multi-center cohorts are therefore essential to confirm the real-world clinical reliability of these metabolic signatures.

Although major chronic comorbidities (e.g., diabetes, hypertension) and the administration of ribavirin were statistically balanced between our clinical groups (*p* > 0.05), acute multi-organ stress and severe hepatic/renal dysfunction induced by CCHFV infection inherently influence systematic amino acid concentrations. While these signatures successfully identify high-risk progression, the overlapping effect of acute physiological stress should be considered during clinical interpretation.

A major methodological challenge in our study is the relatively low number of severe clinical events, specifically ICU admissions (*n* = 18) and fatalities (*n* = 16). Given the quantification of 32 baseline plasma amino acids, this sparse event rate inherently poses a strict risk of overfitting and over-optimistic model performance estimates. To structurally mitigate this risk and enforce dimensionality reduction, we did not fit the final predictive models using the entire amino acid panel simultaneously. Instead, we leveraged rigorous feature selection frameworks—namely Boruta and RFE—alongside penalized modeling approaches such as LASSO to shrink uninformative coefficients and isolate a minimal, high-yield subset of predictors. It also implemented strict internal regularizations, including rigorous feature shrinking via repeated nested cross-validation and survival regularization. Despite these architectural precautions, the exceptionally high performance metrics reported by our optimal configurations (e.g., AUC ~0.95 and survival Concordance Index up to 0.973) should be interpreted with caution. The single-center, retrospective design of this cohort precluded independent external validation. Therefore, multicenter prospective replication in larger independent populations remains a mandatory milestone before these metabolic machine learning pipelines can be translated into routine clinical decision-making tools for CCHF.

In conclusion, this study establishes that advanced machine learning frameworks trained on high-dimensional amino-acid profiles provide an exceptionally accurate and objective system for predicting ICU admission and mortality risk in CCHF patients. By leveraging rigorous feature selection methods such as Boruta and RFE alongside penalized architectures like LASSO and ensemble systems like XGBOOST, the multivariable framework successfully pruned redundant dimensionality to isolate a high-yield predictive metabolic signature. Methodologically, the integration of SMOTE proved crucial in reconstructing minority class decision boundaries, resolving severe class imbalances, and significantly elevating model sensitivity to clinically acceptable standards.

Furthermore, time-to-event architectures like RFSRC and Survival LASSO demonstrated outstanding discriminative capacity, proving the viability of algorithmic risk stratification during the acute phase of hospital follow-up. From a translational medicine perspective, these top-performing configurations transcend traditional univariate statistical approaches and hold immense potential for deployment as automated, web-based clinical decision support tools or electronic health record plugins. Integrating these predictive pipelines into routine intake workflows can instantly generate individualized triage probability scores upon admission, thereby optimizing institutional resource allocation and transforming reactive clinical management into data-driven, proactive healthcare delivery in endemic regions.

## Electronic supplementary material

Below is the link to the electronic supplementary material.

Supplementary Material 1

## Data Availability

The data that support the findings of this study are available on request from the corresponding author. The data is not publicly available due to privacy or ethical restrictions. Data available on request/reasonable request.

## Abbreviations

CCHF: Crimean–Congo Hemorrhagic Fever
CCHFV: Crimean–Congo hemorrhagic fever virus
ICU: Intensive care unit
ML: Machine learning
NK: Natural killer
HPLC: High-performance liquid chromatography
KNN: K-Nearest Neighbors
RFE: Recursive feature elimination
SMOTE: Synthetic minority oversampling technique
RF: Random Forest
LASSO: Least Absolute Shrinkage and Selection Operator
NSC: Nearest Shrunken Centroid
SVM: Support Vector Machine
XGBOOST: eXtreme Gradient Boosting
RFSRC: Random Survival Forest
AUC: Area under curve
ACC: Accuracy
SENS: Sensitivity
SPEC: Specificity
PPV: Positive predictive value
NPV: Negative predictive value
LR+: Positive likelihood ratio
LR-: Negative likelihood ratio
C-index: Concordance index
SGS: Severity Grading Score
SSI: Severity Scoring Index
WBC#: White blood cell count
NEUT#: Neutrophil count
RBC: Red blood cell count
HGB: Hemoglobin
PLT: Platelet count
IG#: Immature granulocyte count
IG%: Immature granulocyte percentage
T.PROT: Total protein
ALP: Alkaline phosphatase
ALT: Alanine aminotransferase
AST: Aspartate aminotransferase
LDH: Lactate dehydrogenase
CK: Creatine kinase
GGT: Gamma-glutamyl transferase
