# Supplementary material for: Machine learning-based prediction of ICU admission and mortality in Crimean–Congo hemorrhagic fever by using wide-range targeted metabolomics
Source: BMC Infect Dis. 2026 Jun 30;26:1334. doi: 10.1186/s12879-026-13787-5 (PMC13371213; doi:10.1186/s12879-026-13787-5)
Supplement: Supplementary file 1 — Supplementary Material 1 [file 12879_2026_13787_MOESM1_ESM.docx]

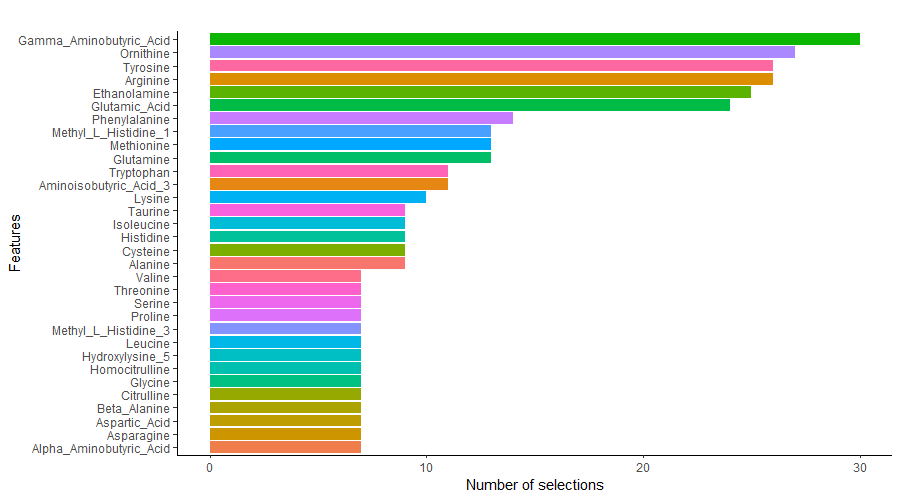


**Figure S1. Feature selection frequency across trials using RFE.**


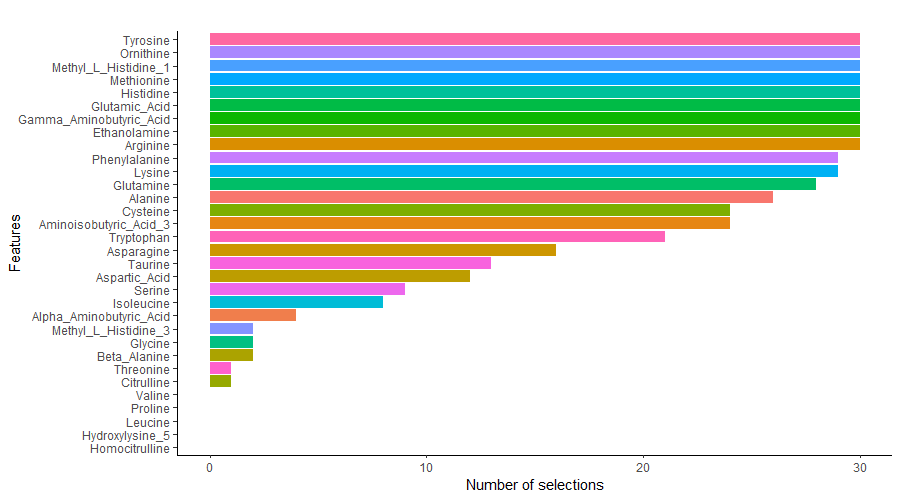


**Figure S2. Feature selection frequency across trials using Boruta.**


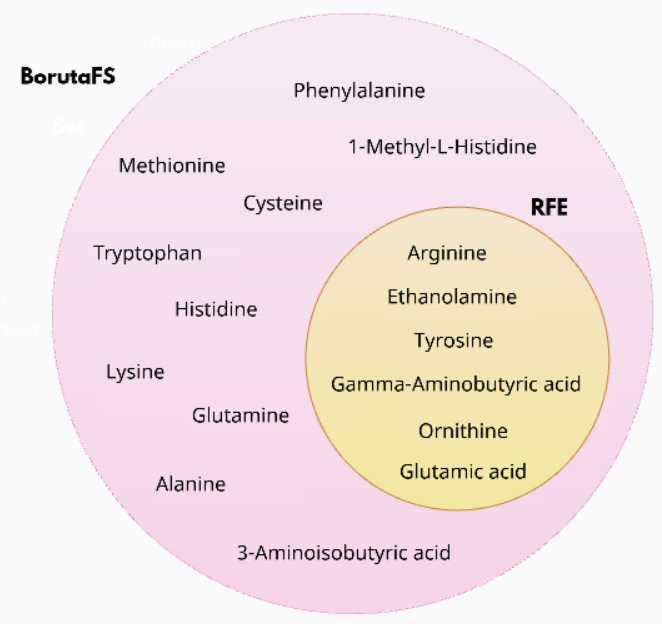


**Figure S3. Features selected in more than 20 trials.**

| **A**  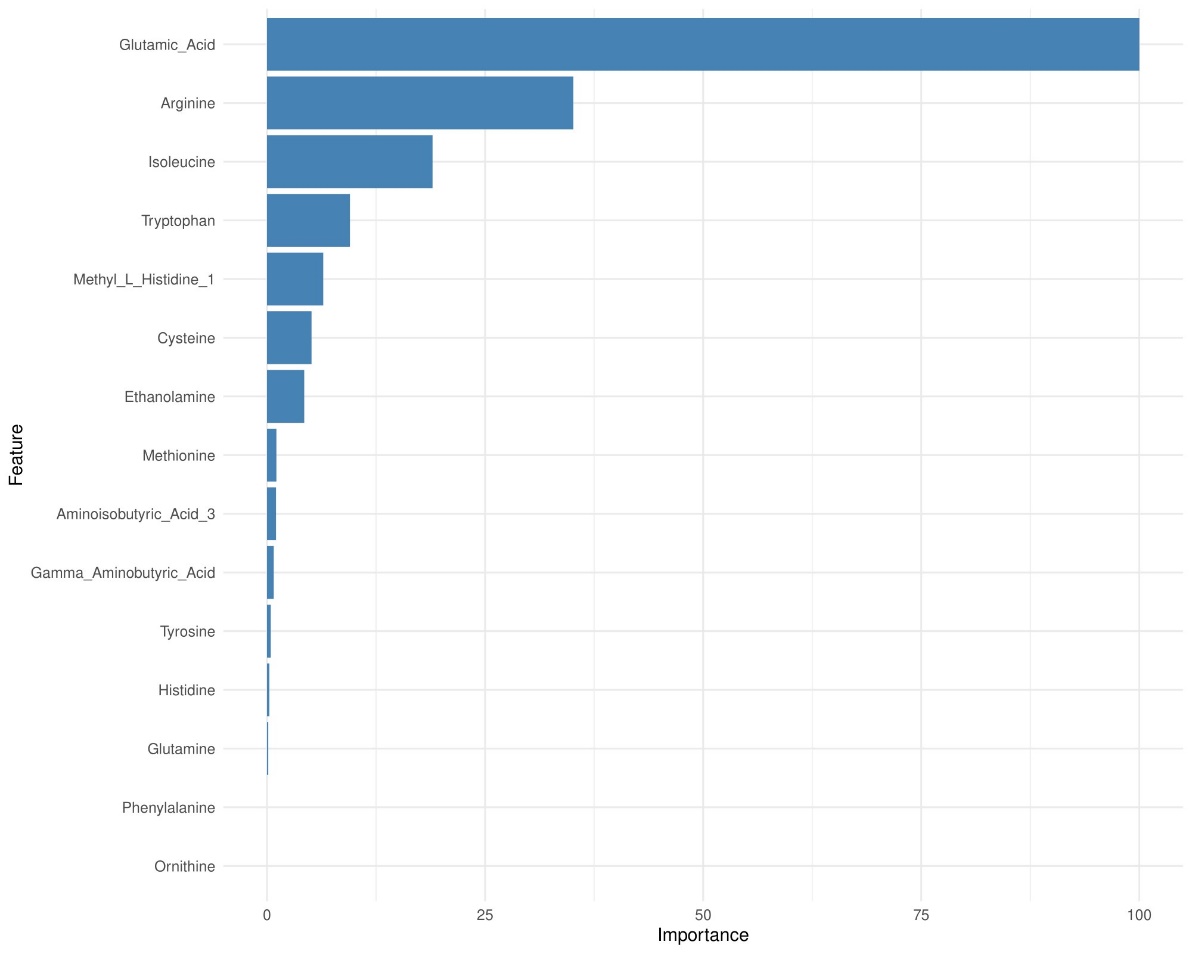 |
| --- |
| **B**  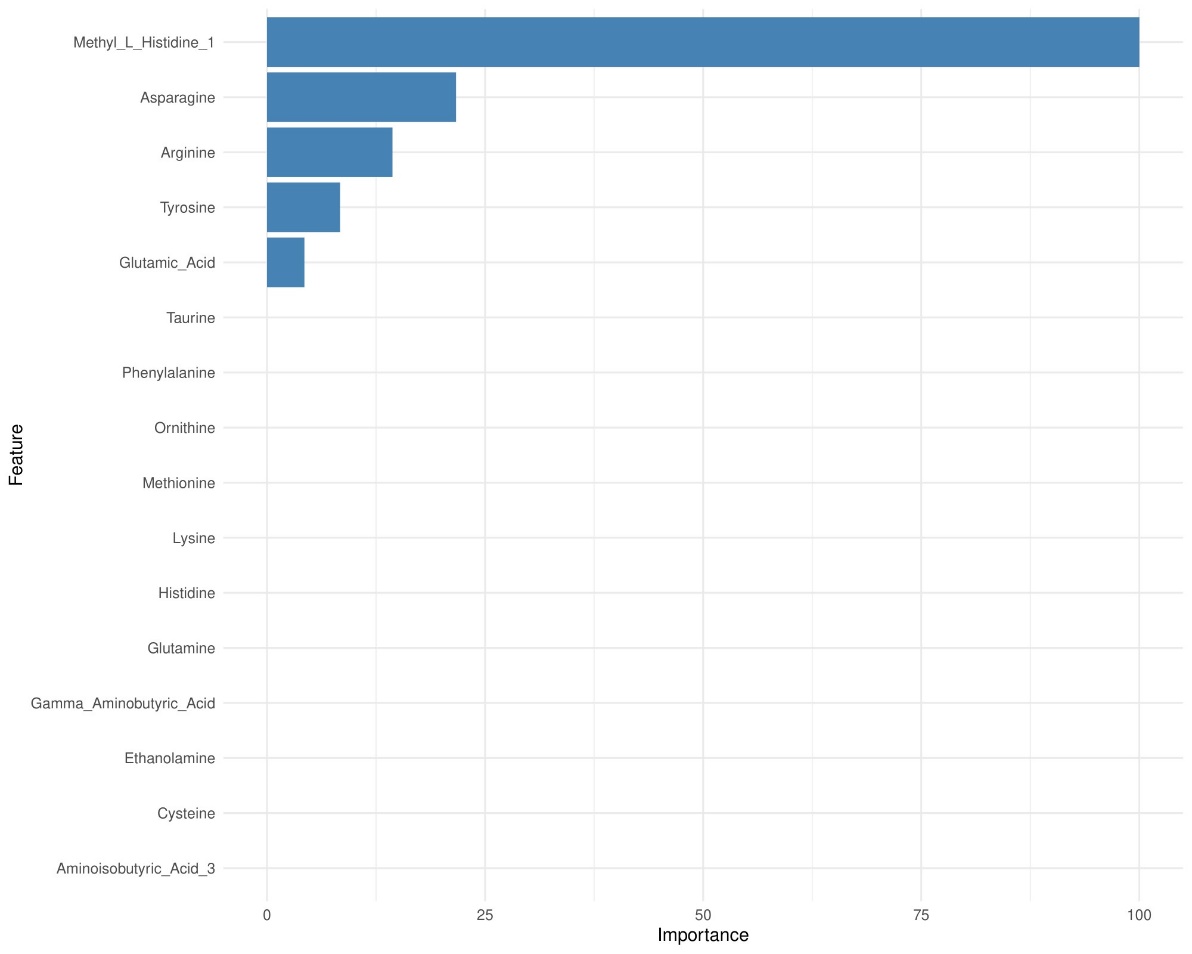 |

**Figure S4. Variable importances on (A) XGBOOST+BORUTA+SMOTE, (B) LASSO+BORUTA+SMOTE.**


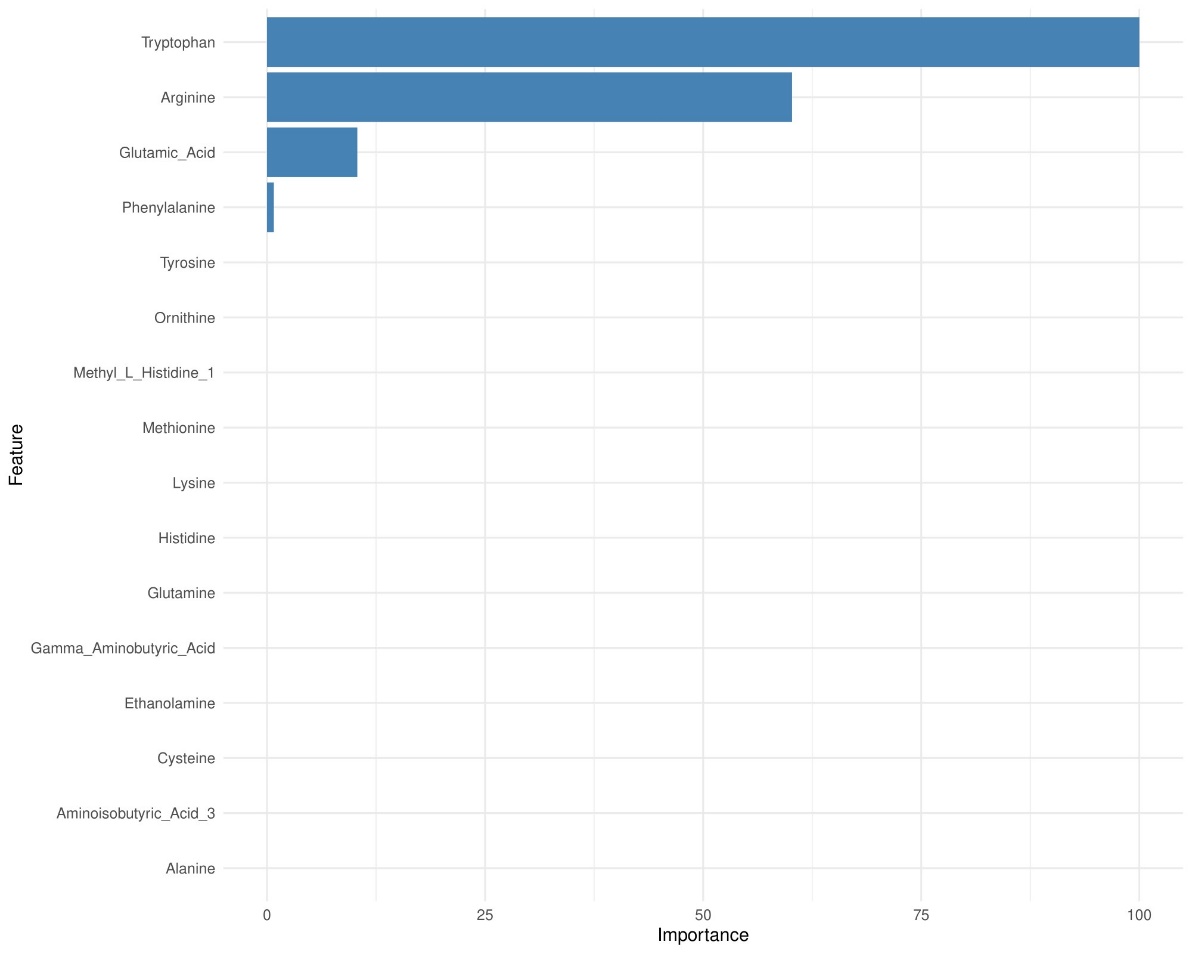


**Figure S5. Variable importances on LASSO+BORUTA+UP.**


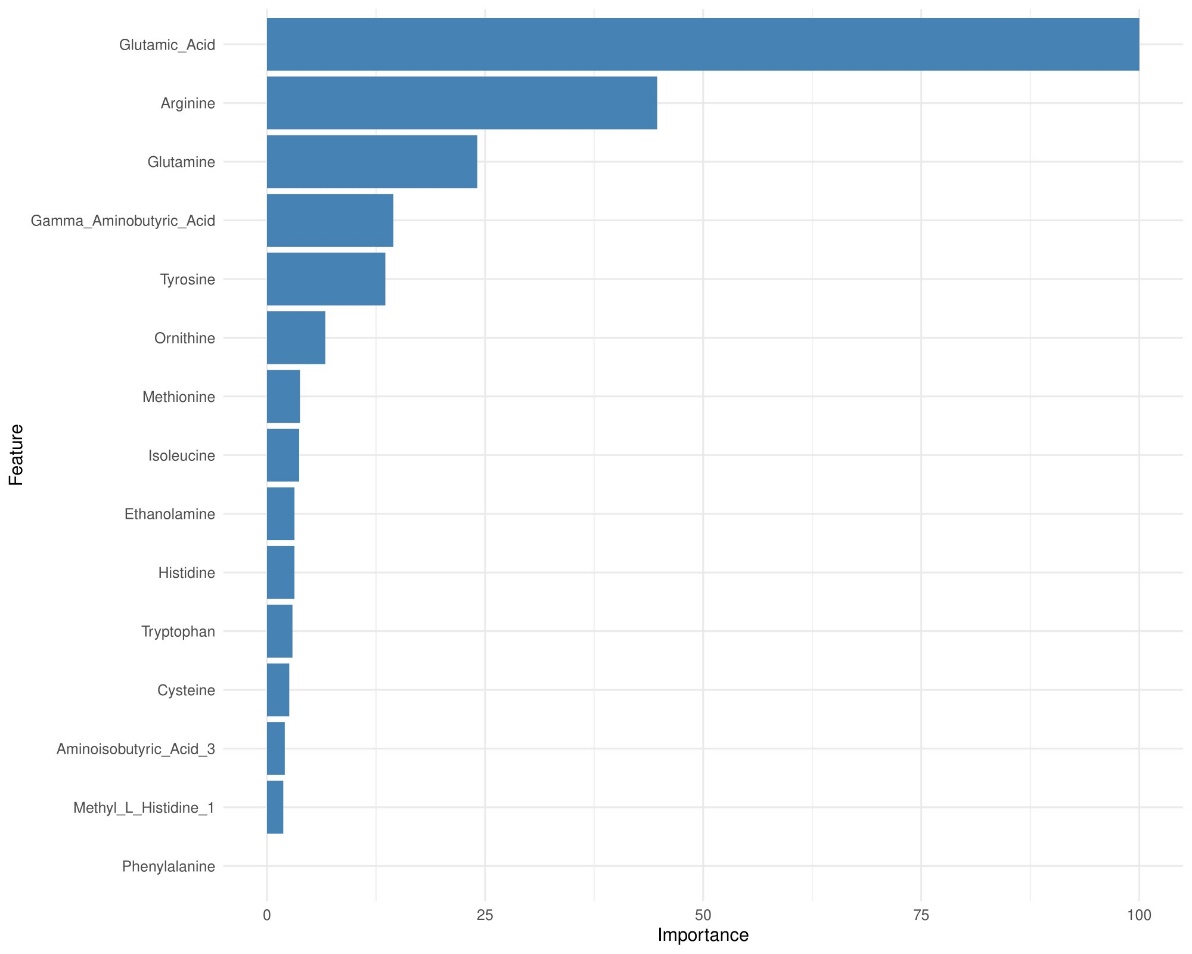


**Figure S6. Variable importances on RF+BORUTA+DOWN.**

**Table S1. Comparison of the predictive performance of the six machine learning models.**

| **Models** | **AUC**  **(95% CI)** | **Accuracy**  **(95% CI)** | **Sensitivity**  **(95% CI)** | **Specificity**  **(95% CI)** | **PPV**  **(95% CI)** | **NPV**  **(95% CI)** | **LR+**  **(95% CI)** | **LR-**  **(95% CI)** |
| --- | --- | --- | --- | --- | --- | --- | --- | --- |
| **RF** |  |  |  |  |  |  |  |  |
| RFE | 0.923(0.783-1) | 0.942(0.926-0.956) | 0.727(0.648-0.797) | 0.98(0.968-0.988) | 0.859(0.786-0.914) | 0.954(0.938-0.967) | 34.84(21.829-55.607) | 0.28(0.216-0.363) |
| RFE+Smote | 0.919(0.769-1) | 0.928(0.911-0.944) | 0.827(0.757-0.884) | 0.946(0.929-0.96) | 0.726(0.652-0.791) | 0.97(0.956-0.98) | 15.18(11.387-20.235) | 0.184(0.13-0.261) |
| RFE+Up | 0.924(0.788-1) | 0.938(0.922-0.952) | 0.754(0.677-0.82) | 0.97(0.957-0.981) | 0.813(0.739-0.875) | 0.958(0.943-0.971) | 25.005(16.939-36.912) | 0.255(0.193-0.337) |
| RFE-Down | 0.936(0.816-1) | 0.905(0.885-0.922) | 0.867(0.802-0.917) | 0.911(0.89-0.929) | 0.629(0.559-0.695) | 0.976(0.962-0.985) | 9.714(7.778-12.131) | 0.147(0.098-0.221) |
| Boruta | 0.938(0.82-1) | 0.945(0.929-0.958) | 0.74(0.663-0.809) | 0.981(0.969-0.989) | 0.868(0.796-0.921) | 0.956(0.941-0.969) | 37.566(23.243-60.717) | 0.266(0.203-0.348) |
| Boruta+Smote | 0.946(0.844-1) | 0.944(0.928-0.958) | 0.84(0.772-0.895) | 0.962(0.947-0.974) | 0.793(0.722-0.853) | 0.972(0.959-0.982) | 21.968(15.608-30.919) | 0.167(0.116-0.241) |
| Boruta+Up | 0.94(0.825-1) | 0.947(0.932-0.96) | 0.787(0.713-0.85) | 0.975(0.962-0.984) | 0.843(0.772-0.899) | 0.964(0.949-0.975) | 30.859(20.259-47.006) | 0.219(0.161-0.298) |
| Boruta+Down | 0.953(0.868-1) | 0.907(0.887-0.924) | 0.84(0.772-0.895) | 0.918(0.898-0.936) | 0.64(0.569-0.707) | 0.971(0.957-0.982) | 10.211(8.084-12.896) | 0.175(0.121-0.252) |
| **LASSO** |  |  |  |  |  |  |  |  |
| RFE | 0.94(0.828-1) | 0.937(0.921-0.952) | 0.667(0.586-0.742) | 0.984(0.973-0.992) | 0.878(0.803-0.932) | 0.945(0.928-0.959) | 41.096(24.147-69.94) | 0.339(0.271-0.425) |
| RFE+Smote | 0.943(0.843-1) | 0.924(0.906-0.94) | 0.854(0.787-0.906) | 0.937(0.918-0.952) | 0.7(0.628-0.765) | 0.974(0.961-0.984) | 13.39(10.281-17.439) | 0.157(0.107-0.231) |
| RFE+Up | 0.933(0.818-1) | 0.903(0.883-0.92) | 0.847(0.779-0.901) | 0.912(0.892-0.93) | 0.626(0.556-0.693) | 0.972(0.958-0.982) | 9.615(7.676-12.043) | 0.169(0.116-0.246) |
| RFE-Down | 0.922(0.791-1) | 0.883(0.862-0.902) | 0.854(0.787-0.906) | 0.888(0.865-0.908) | 0.569(0.502-0.635) | 0.973(0.959-0.983) | 7.593(6.223-9.263) | 0.166(0.113-0.244) |
| Boruta | 0.941(0.855-0.993) | 0.941(0.925-0.955) | 0.694(0.613-0.766) | 0.984(0.973-0.992) | 0.882(0.809-0.934) | 0.949(0.933-0.963) | 42.74(25.148-72.636) | 0.312(0.246-0.397) |
| Boruta+Smote | 0.958(0.876-1) | 0.939(0.923-0.953) | 0.894(0.833-0.938) | 0.947(0.93-0.961) | 0.745(0.675-0.807) | 0.981(0.969-0.989) | 16.76(12.584-22.322) | 0.113(0.071-0.18) |
| Boruta+Up | 0.952(0.861-1) | 0.929(0.912-0.944) | 0.9(0.841-0.943) | 0.934(0.916-0.95) | 0.704(0.634-0.767) | 0.982(0.971-0.99) | 13.627(10.544-17.611) | 0.108(0.067-0.174) |
| Boruta+Down | 0.941(0.841-1) | 0.904(0.884-0.921) | 0.9(0.841-0.943) | 0.904(0.883-0.923) | 0.62(0.552-0.684) | 0.982(0.97-0.99) | 9.358(7.575-11.561) | 0.111(0.069-0.179) |
| **KNN** |  |  |  |  |  |  |  |  |
| RFE | 0.888(0.708-1) | 0.918(0.899-0.934) | 0.554(0.471-0.635) | 0.981(0.969-0.989) | 0.83(0.742-0.898) | 0.927(0.908-0.943) | 28.09(17.172-45.95) | 0.456(0.382-0.545) |
| RFE+Smote | 0.913(0.763-1) | 0.902(0.882-0.919) | 0.834(0.764-0.89) | 0.914(0.893-0.932) | 0.625(0.554-0.693) | 0.97(0.955-0.981) | 9.589(7.636-12.042) | 0.183(0.128-0.262) |
| RFE+Up | 0.86(0.683-0.976) | 0.885(0.864-0.904) | 0.774(0.698-0.838) | 0.904(0.883-0.923) | 0.583(0.512-0.653) | 0.959(0.943-0.971) | 8.041(6.44-10.041) | 0.251(0.187-0.338) |
| RFE-Down | 0.89(0.712-1) | 0.909(0.889-0.926) | 0.7(0.62-0.773) | 0.945(0.927-0.959) | 0.687(0.607-0.759) | 0.948(0.931-0.962) | 12.586(9.378-16.891) | 0.318(0.249-0.406) |
| Boruta | 0.893(0.718-1) | 0.912(0.892-0.928) | 0.5(0.418-0.583) | 0.983(0.972-0.991) | 0.834(0.741-0.904) | 0.919(0.9-0.936) | 28.767(16.991-48.705) | 0.509(0.434-0.598) |
| Boruta+Smote | 0.938(0.829-1) | 0.929(0.912-0.944) | 0.814(0.742-0.873) | 0.95(0.933-0.963) | 0.735(0.661-0.801) | 0.967(0.953-0.978) | 15.953(11.843-21.488) | 0.197(0.141-0.275) |
| Boruta+Up | 0.863(0.682-0.985) | 0.893(0.872-0.911) | 0.74(0.663-0.809) | 0.919(0.899-0.937) | 0.614(0.539-0.685) | 0.954(0.937-0.967) | 9.124(7.15-11.642) | 0.283(0.216-0.371) |
| Boruta+Down | 0.917(0.779-1) | 0.93(0.913-0.945) | 0.694(0.613-0.766) | 0.972(0.958-0.982) | 0.807(0.728-0.871) | 0.948(0.932-0.962) | 23.934(16.033-35.729) | 0.316(0.249-0.402) |
| **NSC** |  |  |  |  |  |  |  |  |
| RFE | 0.92(0.775-1) | 0.903(0.883-0.92) | 0.374(0.296-0.456) | 0.995(0.987-0.999) | 0.919(0.819-0.973) | 0.902(0.881-0.92) | 64.438(26.245-158.211) | 0.631(0.558-0.714) |
| RFE+Smote | 0.922(0.776-1) | 0.933(0.916-0.948) | 0.807(0.735-0.867) | 0.955(0.939-0.968) | 0.757(0.683-0.821) | 0.967(0.952-0.978) | 17.851(13.007-24.497) | 0.203(0.146-0.281) |
| RFE+Up | 0.924(0.783-1) | 0.932(0.915-0.947) | 0.734(0.656-0.803) | 0.967(0.953-0.978) | 0.792(0.715-0.856) | 0.955(0.939-0.968) | 21.823(15.066-31.613) | 0.276(0.212-0.36) |
| RFE-Down | 0.924(0.784-1) | 0.938(0.922-0.952) | 0.754(0.677-0.82) | 0.97(0.957-0.981) | 0.813(0.739-0.875) | 0.958(0.943-0.971) | 25.005(16.939-36.912) | 0.255(0.193-0.337) |
| Boruta | 0.93(0.795-1) | 0.924(0.905-0.939) | 0.514(0.431-0.596) | 0.995(0.987-0.999) | 0.94(0.864-0.98) | 0.922(0.903-0.939) | 88.602(36.467-215.274) | 0.49(0.416-0.577) |
| Boruta+Smote | 0.936(0.815-1) | 0.958(0.944-0.97) | 0.86(0.794-0.912) | 0.975(0.962-0.984) | 0.855(0.788-0.907) | 0.976(0.964-0.985) | 33.736(22.221-51.218) | 0.144(0.097-0.214) |
| Boruta+Up | 0.936(0.812-1) | 0.951(0.936-0.964) | 0.76(0.684-0.826) | 0.984(0.973-0.992) | 0.891(0.824-0.939) | 0.96(0.945-0.972) | 46.849(27.651-79.377) | 0.244(0.184-0.325) |
| Boruta+Down | 0.933(0.805-1) | 0.95(0.935-0.963) | 0.767(0.691-0.832) | 0.982(0.971-0.99) | 0.878(0.81-0.929) | 0.961(0.946-0.973) | 41.353(25.248-67.729) | 0.238(0.178-0.318) |
| **SVM** |  |  |  |  |  |  |  |  |
| RFE | 0.888(0.708-1) | 0.921(0.902-0.936) | 0.72(0.641-0.791) | 0.955(0.939-0.968) | 0.735(0.656-0.805) | 0.952(0.936-0.965) | 15.933(11.541-21.996) | 0.294(0.227-0.38) |
| RFE+Smote | 0.912(0.768-1) | 0.92(0.901-0.936) | 0.687(0.606-0.76) | 0.96(0.945-0.972) | 0.747(0.666-0.817) | 0.947(0.93-0.961) | 16.932(12.027-23.837) | 0.327(0.258-0.415) |
| RFE+Up | 0.921(0.792-1) | 0.92(0.901-0.936) | 0.727(0.648-0.797) | 0.953(0.937-0.966) | 0.727(0.648-0.797) | 0.953(0.937-0.966) | 15.296(11.169-20.948) | 0.287(0.221-0.373) |
| RFE-Down | 0.92(0.783-1) | 0.916(0.897-0.932) | 0.687(0.606-0.76) | 0.955(0.939-0.968) | 0.726(0.645-0.797) | 0.947(0.929-0.961) | 15.195(10.977-21.034) | 0.329(0.259-0.417) |
| Boruta | 0.943(0.838-1) | 0.936(0.919-0.951) | 0.814(0.742-0.873) | 0.958(0.942-0.97) | 0.768(0.694-0.831) | 0.968(0.953-0.979) | 18.971(13.715-26.241) | 0.196(0.14-0.273) |
| Boruta+Smote | 0.946(0.844-1) | 0.927(0.91-0.943) | 0.82(0.75-0.878) | 0.946(0.929-0.96) | 0.724(0.65-0.79) | 0.968(0.954-0.979) | 15.057(11.29-20.081) | 0.191(0.136-0.268) |
| Boruta+Up | 0.947(0.851-1) | 0.931(0.914-0.946) | 0.82(0.75-0.878) | 0.951(0.934-0.964) | 0.741(0.668-0.806) | 0.969(0.954-0.979) | 16.458(12.182-22.234) | 0.19(0.135-0.267) |
| Boruta+Down | 0.947(0.849-1) | 0.927(0.91-0.943) | 0.814(0.742-0.873) | 0.947(0.93-0.961) | 0.727(0.653-0.793) | 0.967(0.953-0.978) | 15.259(11.402-20.422) | 0.198(0.142-0.276) |
| **XGBOOST** |  |  |  |  |  |  |  |  |
| RFE | 0.923(0.791-1) | 0.94(0.924-0.954) | 0.774(0.698-0.838) | 0.969(0.955-0.98) | 0.812(0.738-0.872) | 0.961(0.946-0.973) | 24.719(16.884-36.19) | 0.234(0.175-0.315) |
| RFE+Smote | 0.932(0.822-1) | 0.94(0.924-0.954) | 0.774(0.698-0.838) | 0.969(0.955-0.98) | 0.812(0.738-0.872) | 0.961(0.946-0.973) | 24.719(16.884-36.19) | 0.234(0.175-0.315) |
| RFE+Up | 0.923(0.794-1) | 0.94(0.924-0.954) | 0.774(0.698-0.838) | 0.969(0.955-0.98) | 0.812(0.738-0.872) | 0.961(0.946-0.973) | 24.719(16.884-36.19) | 0.234(0.175-0.315) |
| RFE-Down | 0.914(0.778-1) | 0.94(0.924-0.954) | 0.774(0.698-0.838) | 0.969(0.955-0.98) | 0.812(0.738-0.872) | 0.961(0.946-0.973) | 24.719(16.884-36.19) | 0.234(0.175-0.315) |
| Boruta | 0.944(0.85-1) | 0.944(0.928-0.958) | 0.76(0.684-0.826) | 0.976(0.964-0.985) | 0.845(0.773-0.902) | 0.959(0.944-0.972) | 31.233(20.278-48.105) | 0.246(0.185-0.328) |
| Boruta+Smote | 0.958(0.883-1) | 0.937(0.921-0.952) | 0.867(0.802-0.917) | 0.95(0.933-0.963) | 0.748(0.676-0.81) | 0.977(0.964-0.986) | 16.999(12.661-22.823) | 0.141(0.094-0.212) |
| Boruta+Up | 0.946(0.85-1) | 0.941(0.925-0.955) | 0.794(0.72-0.856) | 0.967(0.953-0.978) | 0.805(0.731-0.865) | 0.965(0.95-0.976) | 23.609(16.357-34.077) | 0.214(0.157-0.293) |
| Boruta+Down | 0.942(0.844-0.998) | 0.861(0.838-0.882) | 0.88(0.817-0.928) | 0.858(0.833-0.881) | 0.518(0.455-0.581) | 0.977(0.963-0.986) | 6.175(5.189-7.348) | 0.14(0.091-0.217) |

**AUC: Area Under Curve, LR+: Positive Likelihood Ratio, LR-: Negative Likelihood Ration, PPV: Positive Predictive Value, NPV: Negative Predictive Value, KNN: K-Nearest Neighboors, NSC: Nearest Shrunken Centroid, RF: Random Forest, SVM: Support Vector Machine, XGBOOST: Extreme Gradient Boosting, RFE: Recursive Feature Elimination.**

**Table S2. Missing Data Summary for specific amino acids or clinical variables.**

| **Group** | **Variable** | **Missing (*n*)** | **Missing (%)** |
| --- | --- | --- | --- |
| Laboratory | ALP | 1 | 0.9 |
| Laboratory | LIPASE | 3 | 2.6 |
| Laboratory | FIBRINOGEN | 1 | 0.9 |
| Laboratory | D-DIMER | 6 | 5.2 |
| Amino acid | Beta-Alanine | 66 | 57.4 |
| Amino acid | Homocitrulline | 11 | 9.6 |

**Table S3. Hyperparameter Tuning Specifications and Search Spaces for Machine Learning Models**

| **Machine Learning Model** | **caret Method** | **Hyperparameter** | **Tested Range / Search Space** | **Optimization / Framework Details** |
| --- | --- | --- | --- | --- |
| Random Forest | Rf | ntree  mtry | 500  5 distict values | Number of trees is set to the package default (ntree = 500). The number of randomly selected predictors (mtry) is dynamically evaluated across 5 values via tuneLength = 5. |
| LASSO Regularization | Glmnet | Mixing percentage (α)  Penalty parameter (λ) | 1  From 0.001 to 0.1 (step: 0.001) | α = 1 enforces a püre LASSO penalty. A grid of 100 sequential regularization values (λ) is evaluated to determine the optimal shrinkage coefficient. |
| K-Nearest Neighbors (KNN) | knn | Number of neighbors (k)  Distance Metric | From 1 to 20 (step: 1)  Euclidean Distance | Evaluates every integer value of k between 1 and 20 inclusive. Standart Euclidean distance is utilized as the default geometric metric. |
| Support Vector Machine (SVM) | svmRadial | Kernel Type  Cost (C)  Sigma (σ) | Radial Basis Function (RBF)  5 distinct values  5 distinct values | Utilizes a non-linear Gaussian/Radial Basis Function kernel. The regularized cost (C) and kernel parameter (σ) are optimized across 5 candidate levels. |
| Nearest Shrunken Centroids (NSC) | pam | Threshold | 5 distinct values | Shrinkage threshold fort he class centroids is optimized automatically across 5 distinct points using a tuneLength = 5 heuristic. |
| XGBoost (eXtreme Gradient Boosting) | xgbTree | nrounds  Learning rate (n)  Maximum depth (max. depth)  Minimum child weight gamma (γ)  Subsample  colsample bytree | 100  0.01, 0.1, 0.2  3, 5, 7  1  0  1  1 | The number of boosting iterations is fixed at 100. A custom tuning grid optimizes shrinkage (0.01, 0.1, 0.2) and tree complexity (max. depth 3, 5, 7) while keeping sub-sampling parameters stable. |
